# Digital Handwriting Kinematics and Physical Performance According to Pentagon-Copy Performance in Community-Dwelling Older Adults: Cross-Sectional Study

**DOI:** 10.2196/85074

**Published:** 2026-05-18

**Authors:** Pau Ferrer-Ramos, Marcos Faundez-Zanuy, Noemí Serra-Payá, Manuel Vicente Garnacho-Castaño, Montserrat Girabent-Farrés

**Affiliations:** 1 Faculty of Psychology, Education and Sport Sciences, Blanquerna Universitat Ramon Llull Barcelona, Catalonia Spain; 2 VITAE Escola Universitària de l'Esport Universitat Abat Oliba CEU Barcelona, Catalonia Spain; 3 Department of Health Sciences, Research Group in Technology Applied to High Performance and Health TecnoCampus Universitat Pompeu Fabra Mataró, Catalonia Spain; 4 TecnoCampus Universitat Pompeu Fabra Mataró, Catalonia Spain; 5 DAFNiS Research Group (Pain, Physical Activity, Nutrition and Health) Campus Docent Sant Joan de Déu Universitat de Vic - Universitat Central de Catalunya (UVic-UCC) Sant Boi de Llobregat, Catalonia Spain; 6 Facultad de Ciencias de la Salud Universidad Internacional de Valencia Valencia Spain

**Keywords:** fine motor control, motor biomarkers, older adults, pentagon-copy test, visuoconstructional performance

## Abstract

**Background:**

Cognitive decline in older adults is often accompanied by subtle motor alterations. Digital handwriting analysis has emerged as a promising noninvasive approach for detecting these changes, but its usefulness in community-based settings remains unclear.

**Objective:**

This study aims to examine the association of handwriting kinematics and physical performance measures with pentagon-copy performance classification in community-dwelling older adults.

**Methods:**

This cross-sectional study included 174 community-dwelling adults aged 60 years or older (mean age 73.9, SD 6.1 years; 108/174, 62% women). Participants completed 10 digital handwriting tasks and a battery of physical performance tests assessing strength, balance, gait, and cardiorespiratory fitness. Group classification was based on pentagon-copy performance and categorized as normal (93/174, 53% participants) or altered (81/174, 46% participants). Adjusted linear regression models included group as the main predictor and age and sex as covariates. Multiple comparisons were controlled using the Benjamini-Hochberg false discovery rate.

**Results:**

After adjustment for age, sex, and multiple comparisons, selected handwriting variables remained significantly associated with altered pentagon-copy performance, whereas no physical performance variables remained statistically significant. The most consistent differences were observed in cognitive effort and mechanical tasks, where participants with altered pentagon-copy performance showed longer contact time (*β*=526.8 ms; *P*<.001) and time on air (*β*=1111.5 ms; *P*<.001), together with lower mean writing pressure (*β*=–2058.8 au; *P*=.003). Overall, group differences were more consistently detected in handwriting-derived variables than in conventional physical performance outcomes after adjusted analyses.

**Conclusions:**

Selected digital handwriting variables, particularly temporal measures, were more consistently associated with altered pentagon-copy performance than physical performance outcomes. These findings suggest that digital handwriting analysis may represent a sensitive complementary approach for exploring subtle functional differences in community-based settings.

## Introduction

The global population is experiencing a marked demographic shift toward aging. According to the World Health Organization [1], the proportion of individuals aged 60 years or older is projected to nearly double, rising from 12% in 2015 to 22% by 2050. Furthermore, it is anticipated that by midcentury, approximately 80% of older adults will reside in low- and middle-income countries.

Aging is intrinsically associated with physiological changes that increase susceptibility to age-related conditions, including cardiovascular disease, musculoskeletal disorders, and neurodegeneration [2], thereby compromising individuals’ functional capacity. To monitor changes in functionality, different tests have been developed to evaluate functional capacity in older adults, with particular emphasis on muscle strength, balance, and overall physical performance. Commonly used tests include handgrip strength, Timed Up and Go (TUG), 6-Minute Walk Test (6MWT), One-Leg Stand Test (OLST), Short Physical Performance Battery (SPPB), countermovement jump (CMJ), and midthigh pull test, all of which have demonstrated reliability and safety in older populations, including those with cognitive impairments [3-6]. Importantly, regular physical activity and structured exercise play a key role in maintaining functional capacity, delaying functional decline, and supporting healthy aging [7].

In parallel with these physical alterations, aging is also characterized by a progressive decline in cognitive function. Cognitive decline encompasses a broad continuum, ranging from normal age-associated cognitive deterioration to pathological dementia [8]. Numerous factors contribute to cognitive decline, with advancing age recognized as the primary independent risk factor, followed by genetic, socioeconomic, and environmental determinants, including nutrition and physical activity [9]. Cognitive functions comprise a range of mental processes integral to acquiring knowledge, processing information, and reasoning, which include perception, memory, learning, attention, decision-making, and language [10]. These domains are essential for the effective execution of activities of daily living.

It is well documented that functional decline is associated with cognitive deterioration in older adults [11,12], and there is evidence that targeted physical exercise interventions may alleviate or reverse these effects [13,14]. Consequently, early detection of functional and cognitive decline is vital for implementing appropriate medical and exercise interventions to slow disease progression. Traditional methods for assessing cognitive decline include verbal and written tests, such as the Mini-Mental State Examination [15], Clock Drawing Test [16,17], Trail Making Test (TMT) [18], and pentagon copying test [17]. Although failure on the intersecting-pentagons task strongly suggests underlying neurocognitive dysfunction, especially in visuospatial and constructional domains, the published literature indicates that the task functions best as a screening marker rather than an isolated diagnostic standard. Studies have shown its usefulness in older adult cognitive screening, in distinguishing Alzheimer disease from healthy aging, and in characterizing visuospatial impairment in dementia with Lewy bodies, while mechanistic work also shows that pentagon-copy errors reflect multifaceted constructional apraxia linked to parietal systems [19,20]. Accordingly, altered pentagon-copy performance can reasonably be interpreted as evidence of probable cognitive vulnerability, but not as sufficient on its own to establish clinical cognitive impairment. Additionally, physical assessments such as gait speed and balance tests have been shown to correlate with cognitive status [21,22].

Handwriting, a complex motor-cognitive task, is notably affected in individuals with neurodegenerative conditions, particularly in text production [23,24], and its kinematic characteristics may provide valuable insights into cognitive status [25,26]. For example, patients with Alzheimer disease and mild cognitive impairment (MCI) often exhibit slower handwriting speeds, increased in-air to on-surface time ratios, prolonged reaction times, decreased fluency, impaired coordination, and reduced consistency in their writing tasks [24,27]. Recent findings have also demonstrated that combining handwriting analysis with gait parameters enhances the classification accuracy of individuals with cognitive impairments [23].

While existing literature has predominantly focused on identifying behavioral markers and cognitive tests associated with cognitive decline, there remains a significant gap in research regarding the detection of cognitive deterioration through simple, noninvasive, and time-efficient motor assessments, such as handwriting analysis and physical performance tests.

Therefore, the aim of this study was to examine whether handwriting kinematic and physical performance variables differ by pentagon-copy performance classification in community-dwelling older adults. We hypothesized that altered pentagon-copy performance would be associated with poorer physical performance and less efficient handwriting kinematics.

## Methods

### Study Design and Population

We conducted a cross-sectional study of community-dwelling participants aged 60 years or older. Participants with a negative medical recommendation to exercise were excluded from the study. A total of 174 participants (age: 73.88, SD 6.05 years; weight: 70.93, SD 13.07 kg; height: 159.07, SD 9.47 cm) divided into men (n=66; age: 73.63, SD 5.96 years; weight: 71.47, SD 13.16 kg; height: 159.56, SD 9.52 cm) and women (n=108; age: 73.90, SD 6.06 years; weight: 70.85, SD 13.10 kg; height: 158.95, SD 9.44 cm), participated in this study.

### Anthropometry and Body Composition

Anthropometric data, including height assessed with a wall-mounted stadiometer (Seca 220) and weight assessed with a scale (Tanita, MC 780-P MA), were collected. Fat-free mass and fat mass were assessed using a bioimpedance analysis system, the BIA 101 Biva Pro (Akern). This provided detailed body composition measurements for the study. Participants were assessed barefoot and in minimal clothing. They were also instructed to refrain from consuming alcohol for 48 hours before the assessments, avoid intense physical activity for at least 12 hours before the assessments, and not wear any metallic objects during the evaluation.

### Handwriting Signal Acquisition

#### Overview

Handwriting signals were recorded using a digitizing tablet and stylus (Wacom Cintiq Pro 16, equipped with the Wacom Pro Pen 2), using an online data acquisition method. This approach represents a significant advancement over traditional offline acquisition, wherein handwriting is performed manually on paper and subsequently digitized through scanning. A principal advantage of online acquisition lies in its capacity to capture pen movements not only during direct contact with the writing surface but also during “in-air” phases, when the pen is lifted and moving above the surface [28]. The tablet operated at a sampling frequency of 100 Hz, recording spatial coordinates (x, y), pen pressure, pen tilt (altitude and azimuth angles), and pen status (pen-up/pen-down). These high-resolution temporal and spatial data allow for a comprehensive characterization of motor behavior during handwriting and drawing tasks.

Participants completed 10 handwriting tasks, each falling into 1 of 3 distinct categories as proposed by Garnacho-Castaño et al [29]: cognitive effort tasks, fine motor control tasks, and mechanical tasks.

#### Cognitive Effort Tasks

This category involves tasks that require substantial mental effort to replicate a complex figure or pattern. Successful completion necessitates working memory, spatial planning, and attentional control to remember the sequence of elements that have already been drawn and to anticipate those yet to follow. Such tasks are often particularly challenging for individuals with cognitive impairments [17], including MCI or early-stage dementia. Included in this category are the pentagon copying test (task 1) and the House Drawing Task (task 2).

#### Fine Motor Control Tasks

Fine motor control tasks, while not demanding in terms of cognitive processing, require precise motor execution. The visual patterns are generally simple and easily memorized after brief exposure; however, successful performance hinges on neuromotor control and sensorimotor integration. Tasks in this category include the Archimedean spiral (task 3), straight-line trace (task 4), spring trace (task 5), and concentric circles (task 6). These tasks are commonly used in neurological assessments and have demonstrated diagnostic value in conditions such as Parkinson disease, where subtle irregularities in amplitude, velocity, and timing of movement serve as early markers of dysfunction [30].

In certain tasks (eg, concentric circles), participants are instructed to trace a preprinted shape with minimal deviation, thereby assessing spatial fidelity and tremor control. Other tasks, such as the continuous line or spring patterns, emphasize rhythmicity, temporal consistency, and trajectory maintenance. The ability to sustain smooth and uniform movement represents an important marker of neuromotor function [31].

#### Mechanical Tasks

Mechanical tasks are characterized by low cognitive demands and involve automatic, overlearned motor patterns commonly used in daily routines. Examples include producing an individual’s signature (tasks 7 and 10), writing capital letters (task 8), and copying a phrase (task 9). These tasks are generally straightforward for healthy individuals and are frequently used in biometric identification systems because of their high consistency and user specificity [32]. Given their automatic nature, deviations in task execution may be more easily attributed to motor abnormalities rather than cognitive deficits [33].

**Figure 1:**
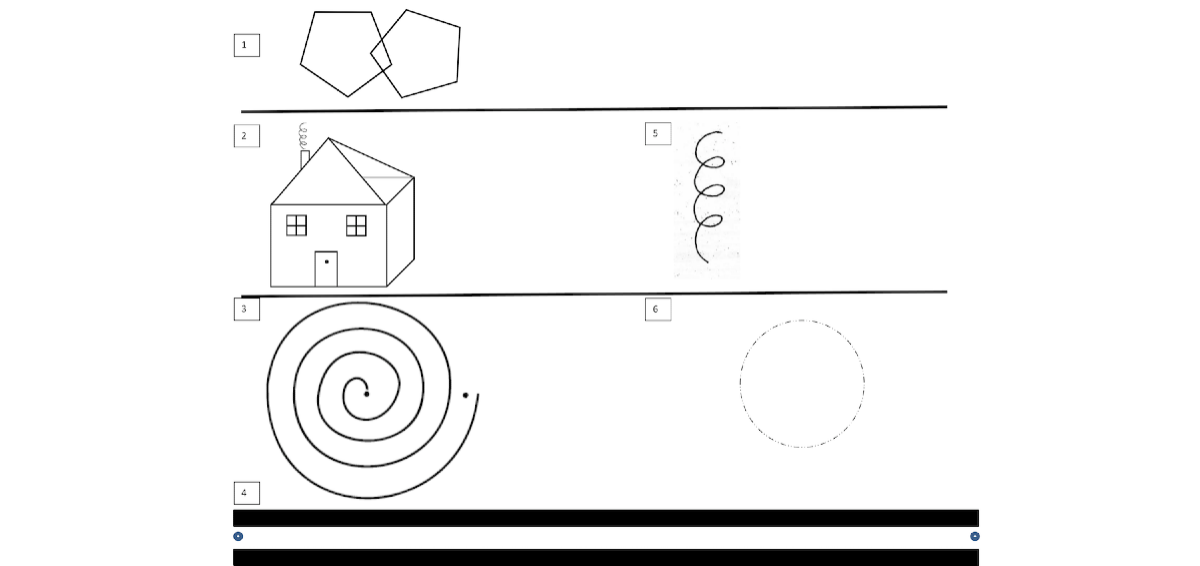
Drawing task sheet template, each identified by a number from 1 to 6.

**Figure 2:**
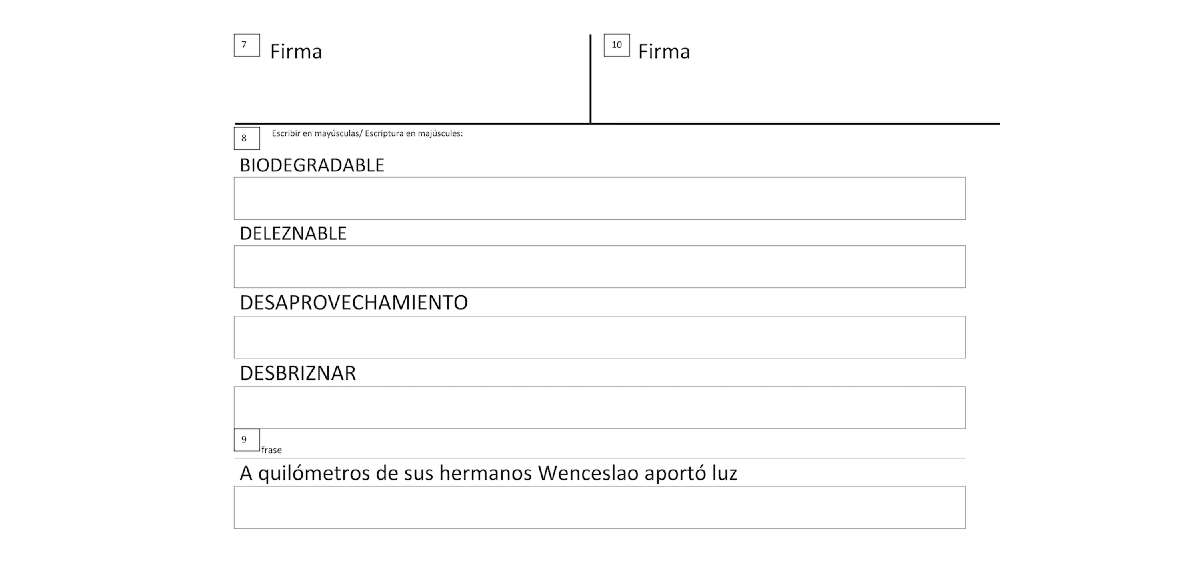
Writing task sheet template, each identified by a number from 7 to 10.

The sheet templates used for each of the 10 tasks are shown in Figures 1 and 2.

### Handwriting Feature Extraction

The main variables obtained from each handwriting task were as follows:

#### Time on Air

Measured in milliseconds (ms) and refers to the total amount of time during which the pen is not in contact with the tablet surface, typically when the hand moves between letters or words. This measure reflects motor planning and writing fluency [34]. Higher values may suggest reduced motor efficiency or greater cognitive demand during the planning of subsequent strokes.

#### Time Down

Measured in milliseconds and refers to the total duration during which the pen remains in contact with the tablet surface while writing. This variable is closely associated with writing speed and the sustained pressure exerted during the task. Higher values may indicate a slower, more controlled writing process, often requiring greater cognitive or motor effort, whereas lower values may reflect a more automated and fluent execution.

#### Mean and Maximum Pressure

Measured in arbitrary units (au) and refer to the average and peak levels of pressure applied by the pen while in contact with the tablet surface. Mean pressure indicates the general force exerted throughout the writing task, whereas maximum pressure reflects the highest force recorded during a single stroke. These parameters are indicative of fine motor control and the physical effort involved in writing [35]. Deviations from typical values may be associated with motor rigidity, tremor, or impaired ability to modulate pressure during handwriting.

#### Mean and Maximum Speed

Measured in mm/s and refer to the average and highest velocities reached by the pen during the writing process, typically measured as the distance covered per unit of time. Mean speed provides an overall indication of the writing pace, whereas maximum speed captures brief moments of rapid movement. These measures reflect the general rhythm and fluidity of handwriting. Alterations in speed may indicate motor slowing or, conversely, impulsive motor patterns characterized by sudden bursts of high velocity.

#### Mean and Maximum Acceleration

Measured in mm/s^2^ and refer to the average and highest rates of change in the pen’s velocity during the writing task. Mean acceleration reflects the general smoothness and consistency of pen movement, whereas maximum acceleration captures sudden, sharp changes in speed. These variables are closely linked to movement coordination and motor control. Elevated or irregular acceleration values may be indicative of tremors, spasmodic movements, or poor regulation of handwriting dynamics.

### Group Classification Based on Pentagon-Copy Performance

To assess classification, the pentagon copying test from the Montreal Cognitive Assessment [36] was administered. In this task, participants were instructed to replicate a specific geometric figure, a pentagon. The test evaluates visuospatial abilities, executive functioning, and attention. Participants were provided with a digital sheet featuring a predrawn pentagon and instructed to replicate the figure by connecting its vertices using straight lines. The task aimed to ensure the accurate completion of the shape in accordance with the specified guidelines.

The test was scored based on the accuracy of the drawing and adherence to the instructions. Errors such as incorrect line placement, additional lines, or failure to follow instructions may indicate impairments in visuospatial processing and executive functioning, which are cognitive domains commonly affected in neurological conditions. Most experts assign a binary score of 0 or 1, with a correct response defined as producing 2 pentagons, each with 5 sides, and a correctly intersected region between them. If these criteria are not met, the score is 0. This scoring process is both straightforward and efficient.

In some contexts, a more granular scoring approach is used, particularly in computerized versions of the test. For example, Nagaratnam et al [19] proposed a scoring system ranging from 1 to 10 based on the quality of the drawing. In line with the original criteria, factors such as figure rotation and tremor were disregarded. In this study, a binary scoring system was adopted because the aim was to classify participants according to pentagon-copy performance rather than to perform a detailed visuoconstructive assessment. Importantly, pentagon-copy performance was used as a task-specific grouping variable and not as a diagnostic criterion or cognitive screening reference standard for cognitive impairment.

The same database used in this study was previously used to investigate cognitive impairment detection through a frontal camera during handwriting tasks, where we reported up to 83% accuracy based on head movement analysis, highlighting the potential of this approach as an automatic assessment method [37].

### Strength Assessment

To evaluate muscular strength and power, a series of standardized tests targeting both upper- and lower-body performance were conducted. These tests included measures of isometric, dynamic, and explosive strength, as described below.

The handgrip strength test was used as a measure of muscular strength [38], particularly for assessing overall upper-body strength. Participants were instructed to grip a Jamar hand dynamometer (Patterson Medical) with maximal force for several seconds. The test was performed on both hands, and the highest value, measured in kilograms, was recorded as the result.

The 30-second arm curl test was used to evaluate upper-body strength and endurance [39]. Participants performed as many bicep curls as possible within 30 seconds using a 2.3 kg dumbbell for women and a 3.6 kg dumbbell for men, with the total number of repetitions recorded as the score.

The midthigh pull test was used to assess lower-body strength [40]. For this test, participants gripped a fixed bar connected to a strength sensor (Chronojump) positioned at midthigh height while maintaining a partial squat posture. They were instructed to pull upward with maximal effort for 3 to 5 seconds, with the peak force generated, measured in Newtons, recorded as a measure of lower-body strength.

The CMJ was used to dynamically assess lower-body explosive strength [41]. Participants started from an upright standing position, performed a rapid downward movement, and immediately jumped vertically as high as possible. Jump height (cm), flight time (ms), and power output (W/kg) were recorded using a force platform (MuscleLab). Each participant performed 3 trials with a 1-minute rest interval, and the highest value was used for analysis.

### Cardiorespiratory Assessment

Cardiorespiratory fitness was evaluated using the 6MWT [42]. The test was conducted on a hardened surface along a 30-m straight-line circuit. Participants were instructed to walk at their fastest pace, without transitioning to running, to cover the greatest possible distance within 6 minutes. They wore comfortable clothing and footwear and were allowed to rest or stop if necessary. To objectively measure ventilatory parameters, participants were equipped with a portable gas analyzer (K5 COSMED). Peak oxygen (peak VO₂, mL/kg/min) uptake was recorded as an indicator of cardiorespiratory fitness, alongside peak carbon dioxide (peak VCO₂, mL/kg/min) production and pulmonary ventilation (VE; L/min) to assess ventilatory efficiency.

### Functional Test and Balance Stability Assessment

To evaluate participants’ functional mobility, balance, and strength, a series of standardized tests were used, including the TUG test, 400-m walking test, and SPPB. These assessments are designed to measure key aspects of physical performance, such as gait speed over short and medium distances, balance, and the ability to perform dynamic movements. The results from these tests help classify individuals based on their functional abilities and identify potential areas of decline in mobility and stability.

The TUG test involves measuring the time required for an individual to rise from a chair, walk 3 m as quickly as possible, turn around, and return to sit down. A completion time of less than 10 seconds is generally considered to be within normal limits. The onset of functional decline is indicated by times ranging from 10 to 20 seconds, although the individual may still be capable of performing basic transfers independently [43].

The 400-m walking test was incorporated within the 6MWT protocol, during which the total time taken by participants to complete a distance of 400 m was recorded. This measurement provides a practical indicator of functional walking capacity and endurance [44].

The SPPB comprises 3 components: a balance assessment, a 4-m gait speed test, and a sit-to-stand test involving 5 repetitions.

The balance assessment evaluates the participant’s ability to maintain specific standing positions for 10 seconds each: feet side by side, semitandem, and tandem. For the feet side by side and semitandem positions, participants received scores of 0 or 1 based on their ability to hold the position (0 points for less than 10 seconds and 1 point for 10 seconds or more). For the tandem position, scoring ranges from 0 to 2 points (0 points for less than 3 seconds, 1 point for 3 to less than 10 seconds, and 2 points for 10 seconds or more) [45]. The total balance assessment score ranged from 0 to 4 points.

Gait speed was measured over an 8-m course, which included 2 m for acceleration, 4 m for timing, and 2 m for deceleration, thereby minimizing early braking bias. Participants were instructed to walk at their usual pace, as if walking on a street. Timing started on the command “Go,” following a 3-second countdown, and concluded as the participant crossed the 4-m mark. Scoring was determined as follows: 0 points if the participant was unable to perform the test, 1 point for times exceeding 6.52 seconds, 2 points for times between 4.66 and 6.52 seconds, 3 points for times between 3.62 and 4.65 seconds, and 4 points for times below 3.62 seconds [45].

The sit-to-stand test involved a straight-backed chair stabilized against a wall. Participants were instructed to cross their arms over their chest and stand up and sit down 5 times as quickly as possible. Timing began from the initial seated position and ended when the participant reached the final standing position after the fifth repetition. The scoring for this component was based on the total time taken to complete the task: 0 points for more than 60 seconds or inability to perform the test, 1 point for between 16.7 and 59 seconds, 2 points for between 13.70 and 16.69 seconds, 3 points for between 11.20 and 13.69 seconds, and 4 points for less than 11.19 seconds [45].

A composite score ranging from 0 to 12 points was derived from all components, categorizing individuals into the following groups: severe limitations (0-3 points), moderate limitations (4-6 points), mild limitations (7-9 points), and minimal limitations (10-12 points) [46].

Static balance was assessed using a force platform (MuscleLab) [47]. Participants stood barefoot on both feet for up to 30 seconds across 3 trials, each separated by a 1-minute rest interval, without external support. The test was performed bilaterally to evaluate balance ability. Center of pressure parameters were recorded, including mean path velocity (mm/s), anteroposterior and mediolateral path velocities (mm/s), anteroposterior and mediolateral oscillation frequencies (Hz), and mean center of pressure displacement (mm).

### Statistical Analysis

Continuous variables were expressed as mean (SD) and categorical variables as frequencies (%). Initial between-group comparisons were performed using 2-tailed Student *t* tests or Mann–Whitney U test, as appropriate, and chi-square tests for categorical variables.

Adjusted analyses were conducted using linear regression models, including group as the main predictor and age and sex as covariates. Regression coefficients (*β*) with 95% CIs were reported.

Variables with nonnormal distributions were log transformed before analysis.

Given the number of comparisons, the false discovery rate (FDR) was controlled using the Benjamini-Hochberg procedure, and both raw and adjusted *P* values are presented. Statistical significance was set at *P*<.05.

### Ethical Considerations

All participants were informed about the aims and procedures of the study and provided written informed consent before participation. The study protocol was approved by the Ethics Committee of Universitat TecnoCampus Mataró-Maresme (CEI1/2022) and was conducted in accordance with the Declaration of Helsinki. To ensure privacy and confidentiality, all participant data were anonymized before analysis and stored securely in password-protected files accessible only to authorized members of the research team. No financial or other compensation was provided to participants for taking part in the study.

## Results

### Group Classification

**Figure 3:**
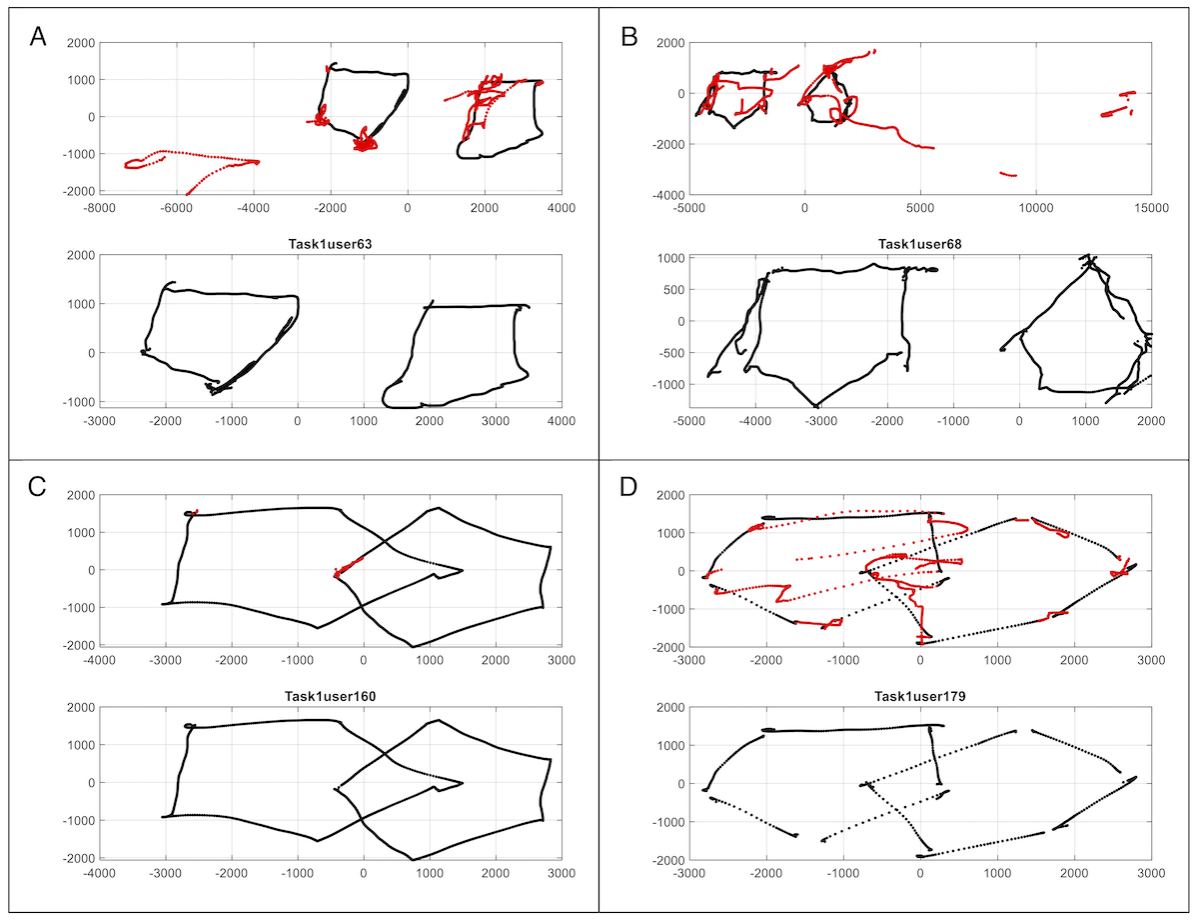
Differences in pentagon-copying test performance between participants with altered pentagon-copy performance (A and B) and those with normal pentagon-copy performance (C and D).

Group classification was based on the results of the pentagon copying test. All drawings were manually reviewed, and participants were classified as either having normal pentagon-copy performance or altered pentagon-copy performance, depending on whether the drawing met the criteria: the presence of 2 pentagons, each with 5 sides, and a correctly formed intersection region between them. Participants who fulfilled these criteria received 1 point; those who did not were given a score of 0. Figure 3 presents the results of this task, illustrating the classification of 4 participants into 2 distinct groups: normal pentagon-copy performance and altered pentagon-copy performance.

Of the total sample, 93 participants were classified as having normal pentagon-copy performance (mean age 69.6, SD 5.9 years) and 81 as having altered pentagon-copy performance (mean age 72.9, SD 5.6 years). Among them, 56.1% (37/66) men and 51.9% (56/108) women were classified as having normal pentagon-copy performance, whereas 43.9% (29/66) men and 48.1% (52/108) women were classified as having altered pentagon-copy performance. Differences in pentagon-copy performance across age groups are presented in Table 1.

**Table 1:** Pentagon-copy performance by age group.

Age group (years)	Normal pentagon-copy performance, n (%)	Altered pentagon-copy performance, n (%)
60-69	57 (69.5)	25 (30.5)
70-79	31 (38.8)	49 (61.3)
≥80	5 (41.7)	7 (58.3)

### Handwriting Performance

After adjustment for age and sex and correction for multiple comparisons using the FDR, several handwriting variables remained significantly associated with group classification based on pentagon-copy performance. The number of statistically significant differences, as well as mean values, regression coefficients, confidence intervals, and *P* values, are presented in Tables 2-5.

**Table 2:** Number of significant differences, after Benjamini–Hochberg correction applied, observed across task types in writing performance variables.

Task type (task numbers)	Total tasks, n	Time on air, n	Time down	Mean pressure, n	Max pressure, n	Mean speed, n	Max speed, n	Mean acceleration, n	Max acceleration, n
Cognitive (T1 and T2)	2	0	2	1	0	0	0	0	0
Fine motor control (T3, T4, T5, and T6)	4	0	0	0	0	0	0	1	0
Mechanical (T7, T8, T9, and T10)	4	2	2	1	0	0	1	0	1

**Table 3:** Statistical values for the variables obtained from the cognitive tasks.

Task and feature		Normal pentagon-copy performance, mean (SD)	Altered pentagon-copy performance, mean (SD)	*β* (regression coefficient)	95% CI	*P* value (row)	*P* value (FDR^a^)
**T1: pentagon copying test**							
	Time on air (ms)	1992.33 (722.49)	2305.01 (919.31)	153.146	–79.80 to 386.09	.20	.43
	Time down (ms)	1044.43 (583.77)	1689.35 (1080.29)	526.799	279.44 to 774.16	<.001^b^	.001^b^
	Mean pressure (au)	10919.99 (4511.14)	8719.79 (4253.78)	–2058.776	–3406.26 to –711.30	.003^b^	.03^b^
	Max pressure (au)	21059.61 (3698.65)	19501.52 (4135.71)	–1326.480	–2524.12 to –128.84	.03^b^	.13
	Mean speed (mm/s)	14.31 (5.82)	13.60 (4.82)	0.173	–1.35 to 1.70	.82	.94
	Max speed (mm/s)	2254.08 (1877.30)	2585.35 (1890.03)	279.486	–272.80 to 831.78	.32	.55
	Mean acceleration (mm/s^2^)	5.51 (3.66)	5.92 (2.71)	0.591	–0.33 to 1.52	.21	.44
	Max acceleration (mm/s^2^)	2255.40 (1877.07)	2587.61 (1888.93)	281.083	–271.38 to 833.55	.32	.55
**T2: house drawing**							
	Time on air (ms)	3806.12 (1574.59)	4192.51 (2126.70)	65.524	–423.79 to 554.84	.79	.93
	Time down (ms)	3282.78 (1219.80)	4402.74 (2214.57)	825.700	316.08 to 1335.32	.002^b^	.02^b^
	Mean pressure (au)	7294.31 (2404.32)	6186.05 (2223.39)	–1015.428	–1735.19 to –295.67	.006^b^	.054
	Max pressure (au)	19921.83 (3515.07)	19152.14 (3546.15)	–526.070	–1637.40 to 585.26	.35	.58
	Mean speed (mm/s)	18.99 (6.04)	17.55 (6.75)	–0.052	–1.82 to 1.72	.95	.97
	Max speed (mm/s)	7105.57 (2395.51)	6524.47 (2450.45)	–556.572	–1269.58 to 156.43	.13	.32
	Mean acceleration (mm/s^2^)	9.47 (4.03)	9.00 (4.20)	0.282	–0.88 to 1.45	.63	.80
	Max acceleration (mm/s^2^)	7103.80 (2397.24)	6524.08 (2449.79)	–555.128	–1268.10 to 157.84	.13	.32

^a^FDR: false discovery rate.

^b^Statistically significant differences.

**Table 4:** Statistical values for the variables obtained from the fine motor control tasks.

Task and feature			Normal pentagon-copy performance, mean (SD)		Altered pentagon-copy performance, mean (SD)	*β* (regression coefficient)		95% CI		*P* value (row)		*P* value (FDR^a^)
**T3: Archimedean spiral**												
	Time on air (ms)	2570.31 (736.70)		2969.46 (1326.04)		294.475	–39.87 to 628.81		.08		.26	
	Time down (ms)	514.92 (458.77)		731.10 (552.95)		164.563	13.99 to 315.14		.03^b^		.14	
	Mean pressure (au)	15562.64 (4110.56)		13690.84 (3740.96)		–1418.389	–2637.34 to –199.44		.02^b^		.11	
	Max pressure (au)	21321.97 (4218.02)		20333.68 (3716.45)		–448.036	–1648.65 to 752.58		.47		.70	
	Mean speed (mm/s)	19.04 (5.90)		18.47 (6.37)		–0.062	–1.99 to 1.86		.95		.97	
	Max speed (mm/s)	8671.91 (2812.86)		9346.66 (2993.25)		707.225	–205.70 to 1620.15		.13		.32	
	Mean acceleration (mm/s^2^)	6.18 (3.84)		6.27 (3.68)		0.099	–1.19 to 1.39		.89		.97	
	Max acceleration (mm/s^2^)	8671.12 (2813.08)		9345.58 (2994.18)		706.648	–206.63 to 1619.92		.13		.32	
**T4: straight-line trace**												
	Time on air (ms)	1350.31 (468.86)		1336.15 (550.89)		–84.357	–236.68 to 67.96		.28		.49	
	Time down (ms)	270.80 (177.89)		356.42 (267.67)		49.522	–20.21 to 119.25		.16		.38	
	Mean pressure (au)	13804.48 (3479.02)		12186.11 (3655.28)		–1198.856	–2216.65 to –181.07		.02^b^		.11	
	Max pressure (au)	19500.58 (3921.30)		18338.06 (4592.50)		–685.930	–1985.92 to 614.06		.30		.52	
	Mean speed (mm/s)	26.47 (8.91)		28.37 (10.66)		3.541	0.50 to 6.58		.02^b^		.11	
	Max speed (mm/s)	8438.09 (3166.12)		9930.53 (4763.90)		1519.761	318.00 to 2721.52		.01^b^		.10	
	Mean acceleration (mm/s^2^)	9.26 (6.86)		11.58 (9.69)		3.034	0.24 to 5.83		.03^b^		.14	
	Max acceleration (mm/s^2^)	8434.92 (3166.60)		9935.58 (4757.37)		1525.121	323.41 to 2726.83		.01^b^		.10	
**T5: spring trace**												
	Time on air (ms)	767.43 (320.16)		910.09 (645.53)		73.060	–77.96 to 224.08		.34		.57	
	Time down (ms)	249.49 (160.02)		313.86 (246.21)		38.755	–28.03 to 105.54		.26		.48	
	Mean pressure (au)	12740.87 (3223.17)		11794.55 (3675.77)		–638.923	–1678.96 to 401.11		.23		.46	
	Max pressure (au)	20484.05 (4003.63)		19176.40 (4471.94)		–836.476	–2099.70 to 426.75		.19		.43	
	Mean speed (mm/s)	33.65 (12.74)		32.56 (16.19)		0.781	–3.99 to 5.55		.75		.91	
	Max speed (mm/s)	13403.85 (1955.36)		13569.41 (3590.10)		43.119	–837.30 to 923.54		.92		.97	
	Mean acceleration (mm/s^2^)	18.53 (7.32)		18.32 (8.88)		0.522	–2.16 to 3.20		.70		.86	
	Max acceleration (mm/s^2^)	13400.57 (1958.30)		13565.90 (3594.64)		42.238	–839.75 to 924.22		.92		.97	
**T6: concentric circles**												
	Time on air (ms)	5559.02 (3275.44)		5166.31 (3737.26)		–976.481	–1995.50 to 42.54		.06		.22	
	Time down (ms)	391.38 (329.59)		605.52 (771.61)		145.142	–15.45 to 305.74		.08		.25	
	Mean pressure (au)	18416.99 (3751.72)		16821.88 (4363.81)		–1263.018	–2526.12 to 0.09		.05		.19	
	Max pressure (au)	23032.83 (3731.70)		21890.59 (4058.39)		–686.131	–1891.81 to 519.55		.27		.49	
	Mean speed (mm/s)	31.59 (18.98)		36.71 (24.46)		8.652	1.96 to 15.34		.01^b^		.09	
	Max speed (mm/s)	3601.41 (1296.68)		4148.01 (1606.90)		414.529	–48.60 to 877.66		.08		.25	
	Mean acceleration (mm/s^2^)	3.59 (2.09)		5.14 (4.26)		1.769	0.75 to 2.79		<.001^b^		.01^b^	
	Max acceleration (mm/s^2^)	3600.78 (1298.13)		4147.52 (1607.63)		414.683	–48.66 to 878.02		.08		.25	

^a^FDR: false discovery rate.

^b^Statistically significant differences.

**Table 5:** Statistical values for the variables obtained from the mechanical tasks.

Task and feature		Normal pentagon-copy performance, mean (SD)	Altered pentagon-copy performance, mean (SD)	*β* (regression coefficient)	95% CI	*P* value (row)	*P* value (FDR^a^)
**T7: signature**							
	Time on air (ms)	667.94 (364.30)	883.74 (551.08)	108.478	–22.64 to 239.59	.10	.29
	Time down (ms)	191.84 (195.47)	337.99 (313.19)	88.176	12.91 to 163.44	.02^b^	.11
	Mean pressure (au)	13161.94 (3818.82)	11722.73 (3735.97)	–741.676	–1898.19 to 414.84	.21	.44
	Max pressure (au)	22306.63 (3949.26)	21314.91 (3355.68)	–353.550	–1460.81 to 753.71	.53	.73
	Mean speed (mm/s)	60.19 (30.81)	49.65 (34.16)	–5.376	–15.12 to 4.37	.28	.49
	Max speed (mm/s)	950.67 (2026.34)	857.06 (1464.04)	–162.247	–633.89 to 309.40	.50	.73
	Mean acceleration (mm/s^2^)	13.92 (10.27)	10.68 (6.47)	–2.059	–4.37 to 0.25	.08	.25
	Max acceleration (mm/s^2^)	859.66 (2056.96)	798.40 (1488.37)	–140.282	–620.54 to 339.98	.57	.77
**T8: capital letters**							
	Time on air (ms)	5456.32 (1682.64)	7146.46 (2923.57)	1111.460	475.21 to 1747.71	<.001^b^	.01^b^
	Time down (ms)	4839.24 (2191.81)	8749.05 (5224.63)	2921.053	1863.79 to 3978.31	<.001^b^	<.001^b^
	Mean pressure (au)	7855.54 (1844.66)	6579.06 (2091.16)	–845.643	–1407.13 to –284.16	<.001^b^	.03^b^
	Max pressure (au)	22000.49 (3758.36)	21329.35 (3435.94)	–115.567	–1179.81 to 948.67	.83	.94
	Mean speed (mm/s)	20.98 (6.44)	17.69 (6.90)	–1.426	–3.28, to 0.43	.13	.32
	Max speed (mm/s)	9060.52 (1802.76)	10252.66 (3050.16)	1166.011	450.70 to 1881.32	.001^b^	.02^b^
	Mean acceleration (mm/s^2^)	10.83 (3.60)	9.45 (3.89)	–.351	–1.41 to 0.71	.52	.73
	Max acceleration (mm/s^2^)	9059.39 (1803.40)	10252.81 (3049.91)	1167.717	452.31 to 1883.12	.001^b^	.02^b^
**T9: phrase copying**							
	Time on air (ms)	3599.74 (939.12)	4840.90 (1885.47)	895.798	475.99 to 1315.61	<.001^b^	.001^b^
	Time down (ms)	3005.53 (1463.13)	5611.05 (3955.06)	2086.101	1228.54 to 2943.66	<.001^b^	<.001^b^
	Mean pressure (au)	8943.97 (2316.99)	7726.09 (2675.64)	–788.751	–1561.70 to –15.80	.05^b^	.18
	Max pressure (au)	22723.55 (3763.68)	22099.78 (3845.61)	–47.616	–1198.17 to 1102.94	.94	.97
	Mean speed (mm/s)	20.41 (5.86)	18.79 (12.41)	0.106	–3.01 to 3.22	.95	.97
	Max speed (mm/s)	6871.78 (4078.34)	7552.58 (4185.74)	984.959	–309.76 to 2279.68	.14	.32
	Mean acceleration (mm/s^2^)	7.62 (3.08)	7.85 (7.17)	1.022	–0.82 to 2.86	.28	.49
	Max acceleration (mm/s^2^)	6869.59 (4078.49)	7550.90 (4184.92)	985.482	–309.06 to 2280.03	.14	.32
**T10: signature**							
	Time on air (ms)	658.56 (356.86)	834.12 (521.77)	82.760	–43.61 to 209.13	.20	.43
	Time down (ms)	280.35 (225.32)	430.60 (356.12)	77.437	1.10 to 153.77	.05^b^	.18
	Mean pressure (au)	11924.60 (3236.74)	10739.14 (3427.03)	–612.123	–1618.27 to 394.03	.23	.45
	Max pressure (au)	22437.45 (3398.14)	21973.26 (3891.51)	–72.172	–1171.64 to 1027.29	.90	.97
	Mean speed (mm/s)	77.26 (43.62)	66.08 (43.97)	–3.623	–16.92 to 9.68	.59	.78
	Max speed (mm/s)	14961.69 (2002.23)	15296.53 (1475.79)	386.022	–114.73 to 886.77	.13	.32
	Mean acceleration (mm/s^2^)	35.23 (22.95)	31.96 (24.13)	0.838	–6.36 to 8.04	.82	.94
	Max acceleration (mm/s^2^)	14958.35 (2013.05)	15292.96 (1476.56)	386.061	–116.01 to 888.13	.13	.32

^a^FDR: false discovery rate.

^b^Statistically significant differences.

The most consistent differences between groups were observed in temporal variables. In particular, time down was significantly higher in participants with altered pentagon-copy performance in tasks 1, 2, 8, and 9. In addition, time on air was significantly higher in this group in tasks 8 and 9.

Significant between-group differences were also found in pressure-related variables. Mean pressure was significantly lower in participants with altered pentagon-copy performance in tasks 1 and 8 after FDR correction.

For kinematic variables, significant differences after FDR adjustment were identified in selected tasks only. In task 6, participants with altered pentagon-copy performance showed significantly higher mean acceleration. In task 8, this group showed significantly higher maximum speed and maximum acceleration.

Overall, the greatest number of significant differences after FDR correction was observed in tasks 8 and 9. No other handwriting variables remained statistically significant after adjustment for multiple comparisons.

### Physical Performance Results

In relation to physical performance tests, the unadjusted analyses showed statistically significant differences between participants with normal and altered pentagon-copy performance in selected variables, including CMJ height and flight time, anteroposterior axis balance stability, and 400-m walking time. However, after adjustment for age and sex, these differences were no longer statistically significant. Likewise, after controlling for multiple comparisons using the FDR, none of the physical performance variables remained statistically significant. No statistically significant differences were observed between the groups in other strength tests, such as handgrip strength, the 30-second arm curl test, or chair stand tests, nor in 4-m gait speed, total distance covered in the 6MWT, the TUG test, or metabolic variables such as peak VO₂. The adjusted results for physical performance variables are presented in Table S1 in Multimedia Appendix 1. The number of participants differed across some tests because certain individuals were unable to complete them.

## Discussion

### Overview

The objective of this study was to examine whether handwriting kinematic and physical performance variables differ according to pentagon-copy performance classification in community-dwelling adults aged 60 years or older. After adjustment for age, sex, and multiple comparisons, only selected handwriting-derived variables remained significantly associated with altered pentagon-copy performance, whereas no physical performance variables remained significant.

The findings of this study should be interpreted within the context of a community-based sample without confirmed clinical diagnoses of cognitive impairment, in which group classification was based on pentagon-copy performance as a marker of task performance potentially related to cognitive vulnerability [20] rather than on a formal neuropsychological or clinical diagnostic assessment. Although failure on the intersecting-pentagons task is generally considered suggestive of underlying neurocognitive dysfunction, particularly in visuospatial and constructional domains, the literature supports its use primarily as a screening marker rather than as an isolated diagnostic standard [19,20]. Accordingly, altered pentagon-copy performance in this study should be interpreted as a marker of probable cognitive vulnerability within a community-based sample, but not as sufficient evidence on its own to establish clinical cognitive impairment. This study was not designed as a cognitive screening validation study; rather, it examined associations between handwriting and physical performance variables and group classification based on pentagon-copy performance in a community-based sample. Within this group-classification framework, differences were observed between individuals with normal and altered pentagon-copy performance in selected kinematic parameters recorded during digital handwriting tasks, whereas no corresponding differences were found in physical performance measures after adjusted analyses. These results are consistent with the hypothesis that early cognitive-related alterations may be reflected in fine motor execution [48], although not in overall physical performance in the present sample, in contrast to previous literature [49].

In the cognitively demanding handwriting tasks, which require mental planning, sustained attention, and working memory, the most consistent differences were observed between the 2 groups. Participants with altered pentagon-copy performance exhibited a significantly prolonged contact time with the screen and a markedly lower mean writing pressure. This pattern may reflect, among other possible explanations, greater disruption in movement planning during the writing process, a finding consistent with previous studies that have documented task execution slowing in complex sequential activities, as well as reductions in applied writing pressure among individuals with cognitive impairment [30,50]. Although velocity values in these tasks were not consistently lower in the altered pentagon-copy performance group, a nonsignificant tendency toward lower mean velocity was observed in some recordings, which may be indicative of less stable writing performance.

In fine motor control tasks, the altered pentagon-copy performance group exhibited higher values in mean acceleration. Contrary to what might be interpreted as an indicator of superior performance, this increase in acceleration likely reflects, among other possible explanations, reduced motor self-regulation and a more impulsive and inefficient execution. Previous evidence suggests that in contexts of cognitive impairment, individuals tend to adopt more erratic motor patterns, characterized by lower temporal and spatial consistency in stroke execution [30]. Higher acceleration in tasks demanding graphical precision may reflect less regulated motor execution.

The mechanically oriented tasks, designed to assess motor execution without additional cognitive demands, also revealed noteworthy differences. In tasks 8 and 9, the altered pentagon-copy performance group demonstrated a significantly longer total execution time (8749 ms vs 4839.2 ms and 5611 ms vs 3005.5, respectively) and higher time on air (7146.5 ms vs 5456.3 ms and 4840.9 ms vs 3599.7 ms, respectively). In addition, the altered pentagon-copy performance group showed lower mean writing pressure (6579.1 au vs 7855.5 au), higher maximum writing speed (10252.7 mm/s vs 9060.5 mm/s), and higher maximum acceleration (10252.8 mm/s^2^ vs 9059.4 mm/s^2^) in task 8. These results suggest reduced motor efficiency even in low-complexity tasks, consistent with previous evidence indicating that cognitive decline may compromise the basal function of motor networks involved in handwriting [27,51], even in the absence of overt clinical signs detectable through conventional neurological assessments.

The findings for physical performance should also be interpreted with caution. Although the unadjusted analyses initially suggested between-group differences in selected variables, including CMJ height and flight time, anteroposterior balance parameters, and 400-m walking time, these associations were no longer statistically significant after adjustment for age and sex. Furthermore, none of the physical performance variables remained significant after correction for multiple comparisons using the FDR, indicating that the initial differences were not robust after statistical adjustment. These findings suggest that, in this community-based sample, conventional physical performance measures may lack sensitivity to detect subtle functional differences associated with pentagon-copy performance classification. Previous studies have identified reduced explosive force production as a robust indicator of cognitively healthy aging [52], and associations have also been reported between MCI and other physical performance measures, including handgrip strength, TUG performance, gait speed, postural control, and cardiorespiratory fitness [53-59]. In this study, however, physical performance measures did not provide support as independent markers of group classification based on pentagon-copy performance. This discrepancy may reflect the relatively subtle nature of the classification approach used, the older age of participants with altered pentagon-copy performance, and other differences in study design, participant characteristics, cognitive reference standards, and severity of impairment across studies. It is also possible that such associations may become more evident in populations with more advanced cognitive impairment.

From a methodological perspective, this study provides a novel contribution by combining noninvasive digital tools for the precise quantification of handwriting kinematics with standardized assessments of physical performance in a community-based sample of older adults. This multimodal approach enabled the simultaneous evaluation of fine motor and functional measures in relation to group classification based on pentagon-copy performance. After adjustment for age, sex, and multiple comparisons, the most consistent associations were observed in selected handwriting-derived variables, particularly temporal measures, whereas physical performance outcomes did not remain statistically significant. In this context, digital handwriting analysis may represent a sensitive and scalable complementary approach for detecting subtle functional differences in community-based settings.

Several limitations should be acknowledged. First, the cross-sectional design does not allow conclusions regarding causality or predictive value. Second, group classification was based on pentagon-copy performance and does not constitute a clinical diagnosis of cognitive impairment. Third, participants with altered pentagon-copy performance were older on average, which may have contributed to the initial unadjusted differences despite statistical adjustment. Fourth, pentagon-copy performance may be influenced by factors other than cognitive status, such as visuoconstructional deficits, fine motor impairment, tremor, or visual difficulties. Finally, the study was conducted in a real-world community setting, where comprehensive neuropsychological evaluation was not feasible.

Future studies should examine these associations using more robust and clinically validated cognitive reference standards, such as the Montreal Cognitive Assessment total score, the Mini-Mental State Examination, or formal neuropsychological assessment. Longitudinal designs will also be needed to determine the predictive value of the handwriting variables identified here and to establish whether these measures may contribute to the development of digital screening tools or automated risk assessment models in primary care and community-based settings.

### Conclusion

In this community-based sample of older adults, only a limited subset of digital handwriting variables remained significantly associated with group classification based on pentagon-copy performance after adjustment for age, sex, and multiple comparisons. The most consistent associations were observed in temporal handwriting measures, particularly time down and time on air, together with selected pressure and kinematic parameters, whereas none of the assessed physical performance variables remained statistically significant after adjusted analyses.

These findings suggest that digital handwriting analysis may be a more sensitive complementary approach than conventional physical performance tests for identifying subtle functional differences associated with altered pentagon-copy performance in older adults. However, because group classification was based on a single visuoconstructional task rather than on a formal clinical or neuropsychological diagnosis, the results should be interpreted with caution and should not be considered evidence of diagnostic screening performance for cognitive impairment.

Overall, this study supports the potential value of digital handwriting analysis as a noninvasive and scalable complementary approach for community-based assessment. Future research should use validated cognitive reference standards and longitudinal designs to determine the clinical relevance, predictive value, and potential application of these handwriting-derived measures in digital screening strategies for older adults.

## Appendix

Multimedia Appendix 1 Physical performance variables according to pentagon-copy performance group. Values are presented as mean (SD), together with adjusted regression coefficients (β), 95% CIs, raw *P* values, and false discovery rate–adjusted *P* values.

## Abbreviations

6MWT: 6-Minute Walk Test
CMJ: countermovement jump
FDR: false discovery rate
MCI: mild cognitive impairment
OLST: One-Leg Stand Test
peak VCO₂: peak carbon dioxide
peak VO₂: peak oxygen
SPPB: Short Physical Performance Battery
TMT: Trail Making Test
TUG: Timed Up and Go
VE: pulmonary ventilation

## Data Availability

The datasets generated and analyzed during the current study are available from the authors upon reasonable request.
