# Supplementary material for: Digital Handwriting Kinematics and Physical Performance According to Pentagon-Copy Performance in Community-Dwelling Older Adults: Cross-Sectional Study
Source: JMIR Aging. 2026 May 18;9:e85074. doi: 10.2196/85074 (PMC13227084; doi:10.2196/85074)
Supplement: Multimedia Appendix 1 [file aging_v9i1e85074_app1.docx]

| Variable | Normal pentagon-copy performance mean (SD) | Altered pentagon-copy performance mean (SD) | β (Group) | 95% CI | *P* value (Row) | *P* value (FDR) |
| --- | --- | --- | --- | --- | --- | --- |
| Handgrip (kg) | 26.53 (8.07) | 25.86 (9.25) | 1.383 | [-.25, 3.02] | .0971 | .6150 |
| Chair stand test (seconds) | 9.70 (2.82) | 9.24 (2.33) | -.747 | [-1.55, .06] | .0693 | .6150 |
| Gait speed (m/s) | 1.03 (.24) | 1.03 (.22) | .039 | [-.03, .10] | .2379 | .6427 |
| CMJ - height (cm) | 9.21 (4.04) | 7.55 (3.77) | -.644 | [-1.71, .42] | .2370 | .6427 |
| CMJ - power (W/kg) | 12.80 (3.08) | 11.92 (3.31) | -.126 | [-1.00, .75] | .7770 | .8750 |
| CMJ - TF (ms) | 267.80 (58.53) | 240.17 (62.92) | -12.387 | [-29.49, 4.72] | .1558 | .6427 |
| Bipodal balance stability - path velocity (mm/s) | 20.33 (3.61) | 21.02 (3.46) | .116 | [-.94, 1.17] | .8289 | .8750 |
| Bipodal balance stability - path velocity AP (mm/s) | 13.32 (2.81) | 13.94 (2.69) | .161 | [-.68, 1.00] | .7081 | .8750 |
| Bipodal balance stability - path velocity ML (mm/s) | 12.52 (2.26) | 12.77 (2.47) | -.020 | [-.70, .66] | .9540 | .9540 |
| Bipodal balance stability - AP (Hz) | .55 (.09) | .58 (.09) | .015 | [-.01, .04] | .3179 | .6427 |
| Bipodal balance stability - ML (Hz) | .60 (.10) | .60 (.09) | .006 | [-.02, .04] | .6894 | .8750 |
| Mean COP distance (mm) | 5.24 (2.56) | 5.06 (1.92) | -.077 | [-.69, .53] | .8059 | .8750 |
| 30 seconds arm curl test (number of repetitions) | 20.40 (5.00) | 20.47 (3.81) | .720 | [-.58, 2.02] | .2770 | .6427 |
| Time up and go test (s) | 7.08 (1.54) | 7.35 (2.04) | -.187 | [-.66, .29] | .4408 | .7544 |
| Mid-thigh pull test (N) | 694.30 (354.11) | 725.11 (386.00) | 80.909 | [-.12, 161.94] | .0503 | .6150 |
| 6 MWT (total distance. metres) | 514.71 (105.89) | 504.01 (80.27) | 9.412 | [-16.50, 35.32] | .4765 | .7544 |
| 400 metres walking test (s) | 270.63 (65.96) | 284.93 (38.88) | 11.532 | [-7.47, 30.53] | .2342 | .6427 |
| VO_2_ peak (mL/kg/min) | 23.04 (7.16) | 22.38 (7.16) | .555 | [-1.64, 2.75] | .6196 | .8750 |
|  | | | | | | |

Supplementary Table S1. Physical performance variables according to pentagon-copy performance group. Values are presented as mean (SD), together with adjusted regression coefficients (β), 95% confidence intervals, raw *P* values, and FDR-adjusted *P* values.
